# Comparison of Target Enrichment Platforms for Circulating Tumor DNA Detection

**DOI:** 10.1038/s41598-020-60375-x

**Published:** 2020-03-05

**Authors:** So Ngo Lam, Ying Chun Zhou, Yee Man Chan, Ching Man Foo, Po Yi Lee, Wing Yeung Mok, Wing Sum Wong, Yan Yee Fung, Kit Yee Wong, Jun Yuan Huang, Chun Kin Chow

**Affiliations:** 1 Department of Research and Development, Medtimes Molecular Laboratory Ltd., Hong Kong, Hong Kong SAR; 2 First Affiliated Hospital of Guangzhou University of Chinese Medicine, Guangzhou, Guangdong China

**Keywords:** Cancer screening, Tumour biomarkers

## Abstract

Cancer-related mortality of solid tumors remains the major cause of death worldwide. Circulating tumor DNA (ctDNA) released from cancer cells harbors specific somatic mutations. Sequencing ctDNA opens opportunities to non-invasive population screening and lays foundations for personalized therapy. In this study, two commercially available platforms, Roche’s Avenio ctDNA Expanded panel and QIAgen’s QIAseq Human Comprehensive Cancer  panel were compared for (1) panel coverage of clinically relevant variants; (2) target enrichment specificity and sequencing performance; (3) the sensitivity; (4) concordance and (5) sequencing coverage using the same human blood sample with ultra-deep next-generation sequencing. Our finding suggests that Avenio detected somatic mutations in common cancers in over 70% of patients while QIAseq covered nearly 90% with a higher average number of variants per patient (Avenio: 3; QIAseq: 8 variants per patient). Both panels demonstrated similar on-target rate and percentage of reads mapped. However, Avenio had more uniform sequencing coverage across regions with different GC content. Avenio had a higher sensitivity and concordance compared with QIAseq at the same sequencing depth. This study identifies a unique niche for the application of each of the panel and allows the scientific community to make an informed decision on the technologies to meet research or application needs.

## Introduction

Cancer is the second leading cause of death worldwide. Cancer-related mortality of most solid tumors remains steady despite intense research on carcinogenesis and significant advancement in effective treatments. It is estimated that 1 in every 6 deaths is related to cancer and late-stage presentation is still very common^1^. For cervical and colorectal cancers, population-wide screenings have contributed to the decreasing mortality^2^. However, there is still an urgent need for accurate, effective and non-invasive paradigms to reduce cancer-related mortality for other common and deadly cancer types via early diagnosis, personalized therapeutic strategies and disease monitoring.

Circulating cell-free DNA (cfDNA) is released into the peripheral blood due to apoptosis, necrosis or active release^3–5^. Higher cfDNA level is found in the plasma of cancer patients comparing to healthy controls^6,7^. Circulating tumor DNA (ctDNA) from cancer cells harboring a unique set of genetic and epigenetic alterations creates a biological signature. This mutation signature is not only specific to cancer in general when compared to normal tissues but is also highly individual specific^3,8,9^. The level of ctDNA, somatic mutations burden, the mutation signature and epigenetic marks are highly correlated with cancer pathophysiology and treatment response^3,10^. Unlike traditional biopsy, profiling of somatic mutations via ctDNA is not only minimally invasive but also provides a less localized and biased sampling. Profiling of ctDNA, released from various cells in the tumor, allows a snapshot of somatic mutation burden to provide a more comprehensive overview of the highly heterogenous tumor^11^. Moreover, for tumors that are not easily accessible with a needle biopsy, ctDNA profiling provides a convenient way to make an informed treatment decision for an optimal outcome. Furthermore, due to the minimally invasive nature of ctDNA profiling, longitudinal examination of the ever-changing mutation signatures which reflect the properties of the evolving tumor, can be used not merely to monitor treatment response and relapse, but also helps clinicians to update therapeutic strategies accordingly^10,12^.

Although the examination of somatic variations in ctDNA is challenging due to the high dilution by cfDNA of normal cell origin in the inherent background, various studies have harnessed the detection power of next-generation sequencing (NGS) via ultra-deep sequencing to uncover these somatic mutations for the development of early screening paradigm, monitoring treatment response and censoring residual diseases^13–18^. Though the sequencing cost has plummeted significantly in the last decade, the need to sequence to high depth to identify rare and highly diluted mutations from the background makes the cost of genome-wide sequencing discouraging for clinical application^19–21^. It is more cost-effective and time-efficient to capture and sequence only the genomic regions which are mutation hotspots or of high clinical importance. To address this need, several companies have developed cancer panels to enrich genomic regions of interest for a specific cancer type or in a pan-cancer manner.

Commercial platforms including Roche’s Avenio ctDNA Expanded panel and QIAgen’s QIAseq Human Comprehensive Cancer panel are currently available for minimal invasive ctDNA detection. These platforms fall into two categories based on their enrichment technologies, probe-based solution hybridization and amplicon-based enrichment. Both platforms are claimed to be applicable for the identification of ctDNA which often have very low allele fraction (AF). While making an educated choice of which commercial platforms and technologies to choose for detection of ctDNA to suit specific application or research needs, several factors including the price, size and design of the region of interest, sensitivity, accuracy and sequencing uniformity are worth careful considerations. However, most of these questions are still left unanswered. In this study, we compared the performance of these two commercially available cancer panels for efficacy in ctDNA detection. We evaluated several key parameters, including (1) panel coverage of clinically relevant variants; (2) target enrichment specificity and sequencing performance; (3) the sensitivity; (4) concordance and (5) sequencing coverage.

## Results

### Overview of platform difference

**Figure 1:**
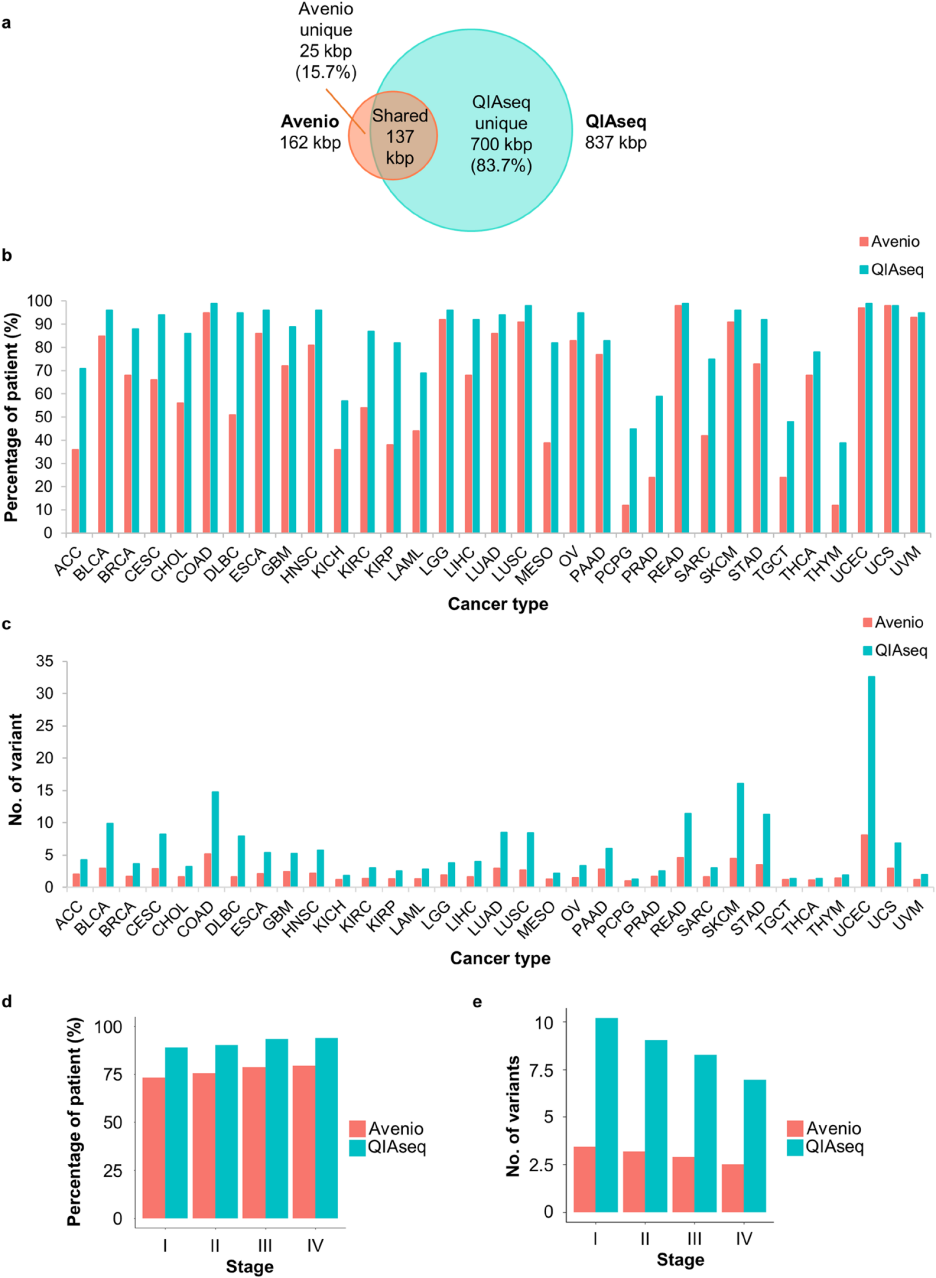
Panel design coverage of somatic mutation in pan-cancer. (**a**) Venn diagram demonstrating the panel size and overlapping targeted region. Panel design coverage on (**b**) the percentage of patient with at least 1 somatic mutation that is detected by Avenio and QIAseq for all cohorts from TCGA at all stages, (**c**) the number of variant per patient, (**d**) the percentage of patient that is covered by the panels at different stage and (**e**) the number of variants per patient in each stage. TCGA study abbreviations, **ACC**: adrenocortical carcinoma; **BLCA**: bladder urothelial carcinoma; **BRCA**: breast invasive carcinoma; **CESC**: cervical squamous cell carcinoma and endocervical adenocarcinoma; **CHOL**: cholangiocarcinoma; **COAD**: colon adenocarcinoma; **DLBC**: lymphoid neoplasm diffuse large B-cell lymphoma; **ESCA**: esophageal carcinoma; **GBM**: glioblastoma multiforme; **HNSC**: head and neck squamous cell carcinoma; **KICH**: kidney chromophobe; **KIRC**: kidney renal clear cell carcinoma; **KIRP**: kidney renal papillary cell carcinoma; **LAML**: acute myeloid leukemia; **LGG**: brain lower grade glioma; **LIHC**: liver hepatocellular carcinoma; **LUAD**: lung adenocarcinoma; **LUSC**: lung squamous cell carcinoma; **MESO**: mesothelioma; **OV**: ovarian serous cystadenocarcinoma; **PAAD**: pancreatic adenocarcinoma; **PCPG**: pheochromocytoma and paraganglioma; **PRAD**: prostate adenocarcinoma; **READ**: rectal adenocarcinoma; **SARC**: sarcoma; **SKCM**: skin cutaneous melanoma; **STAD**: stomach adenocarcinoma; **TGCT**: testicular germ cell tumors; **THCA**: thyroid carcinoma; **THYM**: thymoma; **UCEC**: uterine corpus endometrial carcinoma; **UCS**: uterine carcinosarcoma; **UVM**: uveal melanoma.

Although both panels aim for the detection of somatic mutations via deep sequencing, the development, design and technologies of the panels and kits are substantially different. Avenio is a commercialized kit developed from CAPP-seq which enriches recurrent mutations in driver genes via hybridization to DNA probes^16,22,23^. On the other hand, QIAseq enriches target regions through PCR amplification with a proprietary single primer extension reaction (Table 1). The two panels were 162 kbp (Avenio) and 837 kbp (QIAseq) in size with around 136.7 kbp in common. 15.7% and 83.7% of the panels are unique to Avenio and QIAseq respectively (Fig. 1a).

**Table 1:** Overview of characteristics of Avenio ctDNA Expanded panel and QIAseq Human Comprehensive Cancer panel. *Calculated from the .bed file provided by the manufacturers. ^$^Representation of the relative cost of the kit per reaction.

	Avenio ctDNA Expanded panel	QIAseq Human Comprehensive Cancer panel
Size (kbp)*	162.10	836.67
Enrichment technology	Hybridization	Amplicon
Extraction method	Affinity column	NA
Fragmentation method	NA	Enzymatic
Automation	++	++
Throughput	++	+++
Flexibility	Custom unavailable; have 3 panel choices	Custom available
Used of UMI	Yes (4 bp)	Yes (12 bp)
Unique dual index compatibility	No	Yes
Cost	$$$	$$

The Avenio ctDNA Expanded kit but not the QIAseq Human Comprehensive Cancer panel includes a cfDNA extraction kit that extracts cfDNA with affinity columns (Table 1). For the fairness of comparison and to ensure differences in the performance of the panels are not due to the use of different cfDNA extraction protocols. We used the cfDNA extraction kit (TIANGEN Biotech Co. Ltd., Beijing, China) to extract all the samples in this study. Though the Avenio cfDNA extraction kit was not used, we compared its extraction yield and DNA size profile with TIANGEN’s cfDNA extraction kit. Both extraction kits extracted cfDNA with comparable quantity (Avenio: 36.89 ± 33.44 ng; TIANGEN: 45.10 ± 28.85 ng; *p* = 0.072; data not shown). Furthermore, Avenio extracted DNA with a size of 173.8 ± 3.3 bp while TIANGEN extracted DNA of 175 ± 2.2 bp (*p* = 0.412; data not shown). The size distribution of DNA extracted by the kits is characteristic to cfDNA^24^.

By far, fresh tumor biopsies and formalin-fixed paraffin-embedded (FFPE) sections are still the most common sample types for molecular mutation profiling. Kits that can process multiple sample types would provide a fairer evaluation of concordance and accuracy of ctDNA detection. The Avenio ctDNA panel is specialized for ctDNA application while the QIAseq panel is also applicable to freshly extracted DNA from tumor samples and FFPE sections. Though Roche also provides Avenio panel for FFPE samples, compatibility of the QIAseq Human Comprehensive Cancer panel with fresh biopsy and FFPE allows the study of concordance of variants detected in ctDNA with tumor biopsy or FFPE using the same kit.

For clinical applications, whether the kit is compatible with robotic automation systems and its maximum number of multiplexing would highly impact the overall cost and economic incentive of marketing the mutation detection test. Both kits use magnetic beads for cleaning up and size selection and are compatible with most library preparation automation systems for NGS. However, since QIAseq consists of 96 indexing combinations for dual indexing, the throughput of QIAseq is much higher than that of Avenio (Avenio: 16 single-index adapters; QIAseq: 96 dual-index adapters). Due to the proprietary single-index adapter design of Avenio, the panel is incompatible with the unique dual index which has been found to mitigate index-hopping in pattern flow cells of Illumina. Furthermore, both panels made use of unique molecular index (UMI) to tag individual DNA fragments to provide a better quantification of copy number and *in silico* error suppression.

### Panel coverage of clinically relevant variants

To test the degree of the panel coverage of recurrent mutations in cancers, we studied the number of mutations each panel can target enrich for detection using variants data of 33 cohorts profiled by The Cancer Genome Atlas (TCGA). We studied the number of patients with at least 1 somatic mutation which is targeted by Avenio and QIAseq. On average, Avenio covered 71% of patients with an average of 3 mutations per patient while 88% of patients have an average of 8 mutations covered by QIAseq (Fig. 1b,  c). Since the number of variants detected depends greatly on panel size, we studied the variant detection efficiency by normalizing the number of variants per patient detected by the panel size of each platform. After normalization, Avenio detected 1.80 variants per 100 kbp of the panel. QIAseq was heavily penalized by its large panel size, the number variants targeted per 100 kbp of the panel was reduced to 0.95.

Despite its much smaller panel size, Avenio has comparable patient coverage with QIAseq for lung (LUAD: Avenio 86% vs QIAseq 94%; LUSC: Avenio 91% vs QIAseq 98%) and colorectal cancer (COAD: Avenio 95% vs QIAseq 99%; READ: Avenio 98% vs QIAseq 99%) (Fig. 1b), which could be due to the use of an iterative algorithm for the design of the selector^22^. On the other hand, QIAseq covered 40% more patients with rare cancers such as kidney cancer (KIRP), lymphoma (DLBC) and mesothelioma (MESC). Overall, QIAseq targeted more variants per patient (Fig. 1c).

Both panels were able to cover mutations frequently occurred in the early stage of cancers (Fig. 1d,  e). Avenio targeted 73% and 76% of stage I and II patients respectively with at least 1 mutation. Consistently, QIAseq showed a higher coverage of these early-stage cancer patients. It covered around 90% of patients at stage I and II.

**Figure 2:**
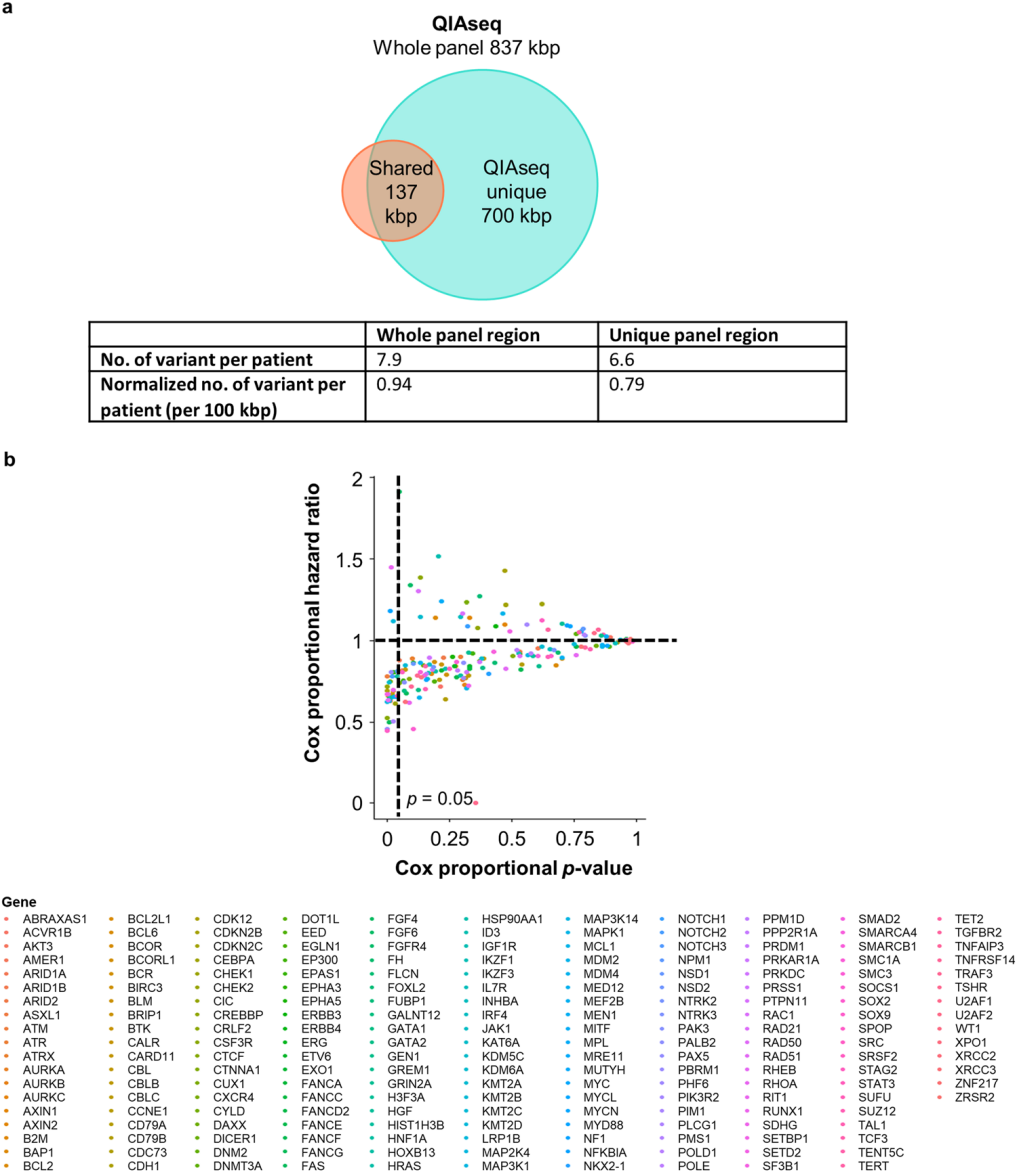
Evaluation of the add-on value of the QIAseq unique region in the detection of clinically significant variants. (**a**) No. of variants per patient detected by QIAseq whole panel region and unique panel region. (**b**) The clinical significance of genes that are detected by the unique panel region of QIAseq for all cohorts from TCGA at all stages.

Although the sequencing cost has decreased substantially over the last decade, due to the need to sequence to ultra-high depth to discover mutations in ctDNA in plasma, a larger panel size would greatly increase the cost and prohibit its application in cancer detection and surveillance. Furthermore, a larger panel would decrease the sensitivity and accuracy via the identification of artifactual mutations which further reduces signal-to-noise ratio^18^. Since QIAseq is more than 5 times larger than Avenio, we investigated the unique region targeted by QIAseq to evaluate its add-on value on the detection of clinically significant variants. On average, this unique region alone would allow the QIAseq panel to target an average of 6.6 variants per patient (Fig. 2a). However, the number of variants targeted per 100 kbp decreased from 0.95 (whole panel region) to 0.79 (unique panel region), suggesting the density of recurring somatic mutations in these genomic regions within the unique panel region is relatively lower (Fig. 2a). Furthermore, the clinical significance of these variants targeted in the QIAseq unique region was analyzed and annotated with the ClinVar database. The ClinVar database annotates different clinical significance values of the variant, including 1) according to the American College of Medical Genetics and Genomics or the Association for Molecular Pathology guidelines; 2) drug response and 3) risk factor. Although over 70% of these variants in the unique region of QIAseq were having a moderate to high impact on the protein function, none has been proved to be correlated with drug response. For variants with moderate or high clinical significance, their impact on survival was further investigated, 41 out of the 204 genes have been shown to present significant impacts on patient survival in cohort having at least one moderate or high clinical variants compared to the wild-type cohort (Fig. 2b).

### Target enrichment specificity and sequencing performance

**Figure 3:**
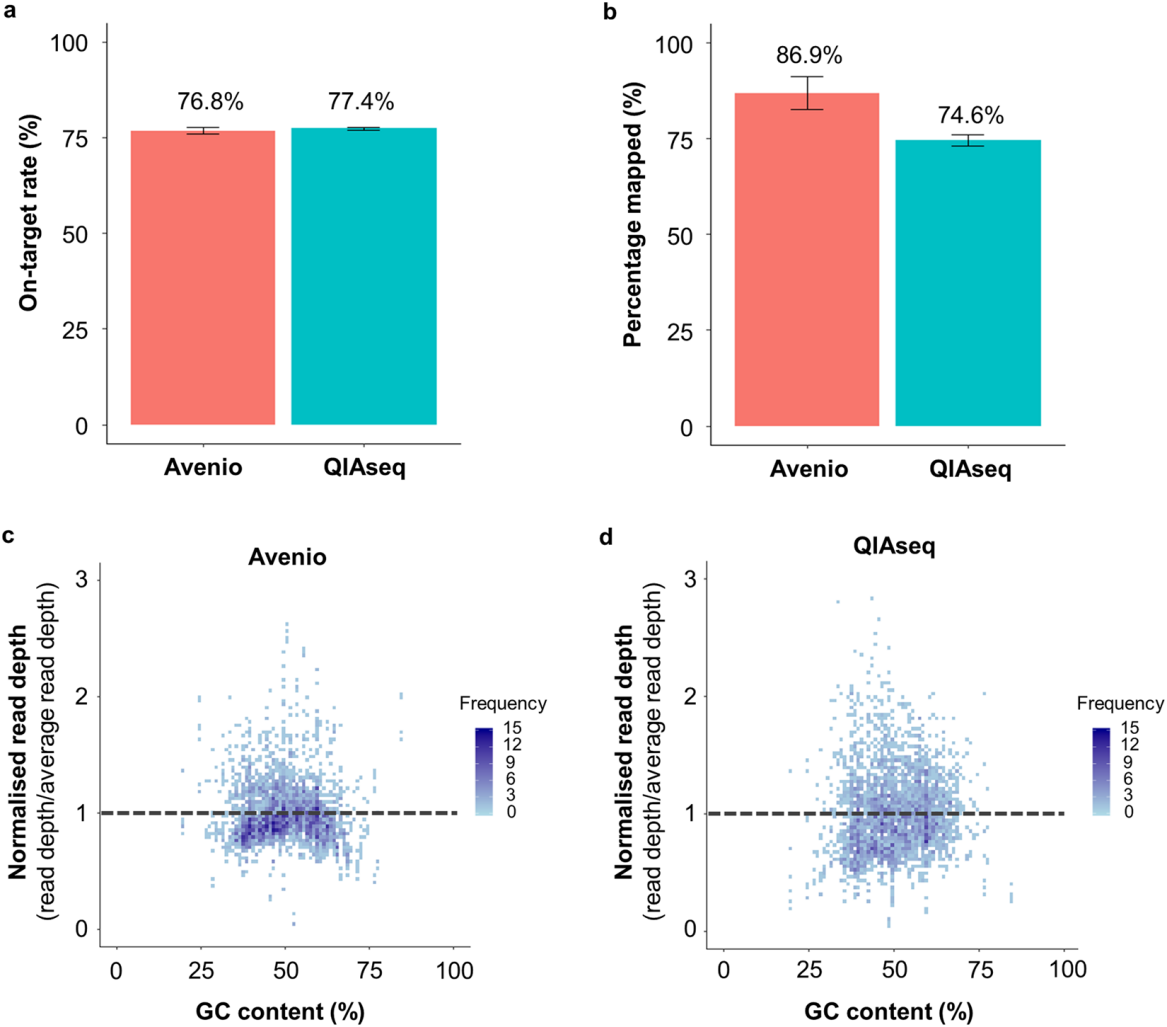
Sequencing performance of the panels. (**a**) On-target rate, (**b**) percentage mapped of the reads. Normalised read depth across reads of different GC content within the shared region of (**c**) Avenio and (**d**) QIAseq.

To assess the extent of target enrichment, the on-target rate, which was defined as the percentage of reads mapped to the target region, was compared. Both panels had comparable on-target rate (Avenio: 76.8 ± 0.9%; QIAseq: 77.4 ± 0.4%) (Fig. 3a). Avenio demonstrated a higher percentage of reads mapped, 87% comparing to 75% of QIAseq (Fig. 3b). GC content of the fragment sequence has been shown to have a systemic effect on target enrichment and sequencing uniformity. We next examined the uniformity of sequencing across genomic regions of different GC content. For a fair comparison, only the shared region of both panels was studied. The number of reads of the fragments within the shared region was further normalized by the average read depth. The heatmaps showed that Avenio had more fragments having a normalized read depth close to 1, suggesting that Avenio had more uniform coverage across regions with different GC content (Fig. 3c,  d).

### The sensitivity of ctDNA detection

**Figure 4:**
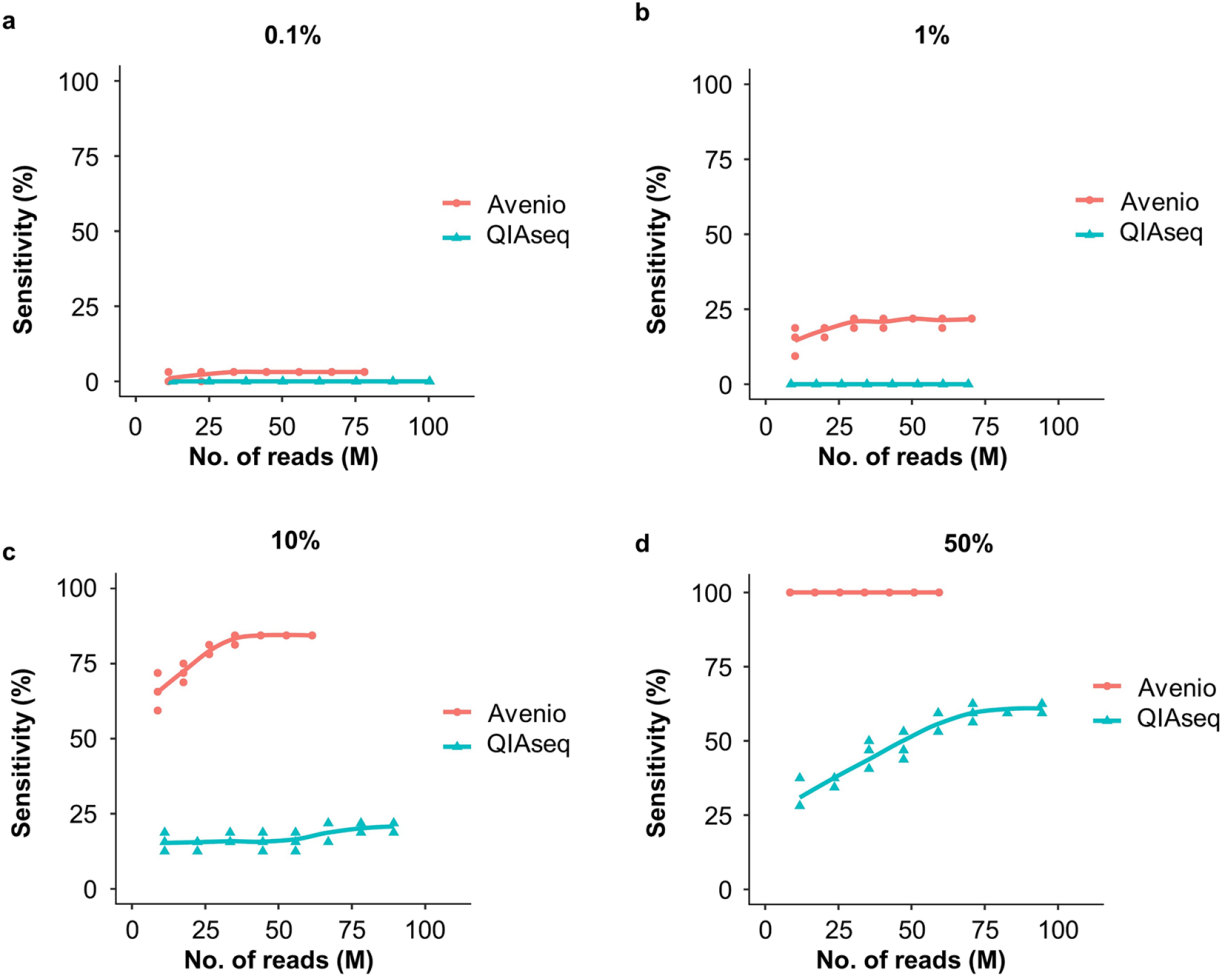
The sensitivity of detecting spike-in variants at different read depth. Libraries prepared with 15 ng of cfDNA spiked in with (**a**) 0.1%, (**b**) 1%, (**c**) 10% and (**d**) 50% of cfDNA reference were sequenced with ¼ lanes of NextSeq High Output kit. Read pairs were normalized by random sampling at 50–350 M read pairs with 50 M increment.

To assess the sensitivity of detecting frequently occurring somatic mutations in each platform, we sequenced libraries generated by spiking 0.1, 1, 10 or 50% of cfDNA reference which consists of 32 well-characterized mutations into the cfDNA of a healthy volunteer of Han Chinese ancestry. These cfDNA references are commonly enriched by both platforms. cfDNA from the healthy volunteer was pre-sequenced with both platforms to ensure the sample is free of the spike-in variants in the background. The library was constructed and enriched in accordance with the manufacturers’ instructions. 150-bp paired-end reads were generated using ¼ lane of an Illumina NextSeq High Output kit. Read counts were normalized between the two platforms by randomly selecting 50–350 million reads with an increment of 50 million reads. For both platforms, the sensitivity of variant detection increased with the number of read pairs (Fig. 4a–d). The sensitivity of variant detection depends greatly on the AF and sequencing depth. With 15 ng of cfDNA input consisting of variants with expected AF of 0.075–2.96% (10% cfDNA reference spiked in), a clinically relevant AF of most variants detected in ctDNA^25,26^, sensitivity of Avenio started to plateau at 85% with 35 million of reads while the sensitivity of QIAseq levelled off at 20% with 65 million of reads (Fig. 4c). With 50% cfDNA reference spike-in, Avenio demonstrated a higher sensitivity of ctDNA detection, 100% at all read depth, compared to around 60% with at least 60 million of reads in QIAseq (Fig. 4d).

### Concordance and accuracy

**Figure 5:**
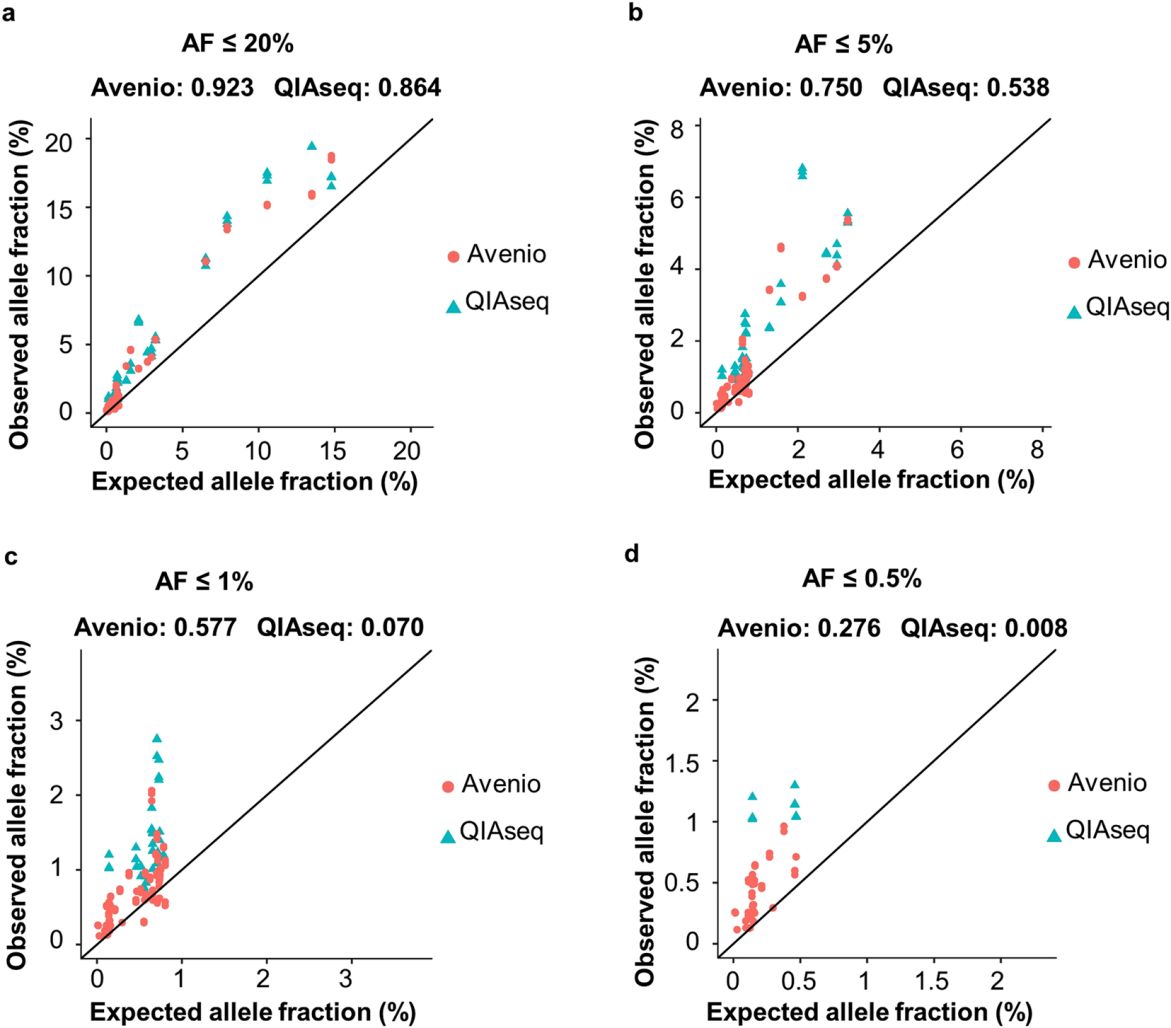
The concordance and linearity of expected AF and observed AF of spike-in variants at expected AF threshold of (**a**) ≤20%, (**b**) ≤5%, (**c**) ≤1% and (**d**) ≤0.5%.

To investigate the accuracy of Avenio and QIAseq in estimating AF of somatic mutations, we computed the concordance correlation coefficient (CCC) between AFs of spike-in references which are independently quantified with ddPCR (expected AF) and the Avenio or QIAseq detected AFs (observed AF) (Fig. 5a–d). ddPCR estimates the absolute copy number of the spike-in references. Thus, it is used as a “gold standard” to evaluate the concordance and accuracy of Avenio and QIAseq. CCC was estimated using U-statistics without making assumption on the AF distribution normality^27^. Concordance between expected and observed AF with 350 million normalized read pairs was 0.923 (95% confidence interval (CI): 0.891–0.946) for Avenio and 0.864 (95% CI: 0.819–0.898) for QIAseq for AF ≤ 20% (Fig. 5a). For AF ≤ 5%, Avenio showed higher accuracy of AF estimation (Avenio: 0.750, 95% CI: 0.678–0.808; QIAseq: 0.538, 95% CI: 0.447–0.619) (Fig. 5b). Furthermore, at a clinically relevant AF, AF ≤ 1%, Avenio demonstrated substantially higher concordance (Avenio: 0.577, 95% CI: 0.541–0.610; QIAseq: 0.070, 95% CI: -0.108–0.244) (Fig. 5c).

### Sequencing coverage

**Figure 6:**
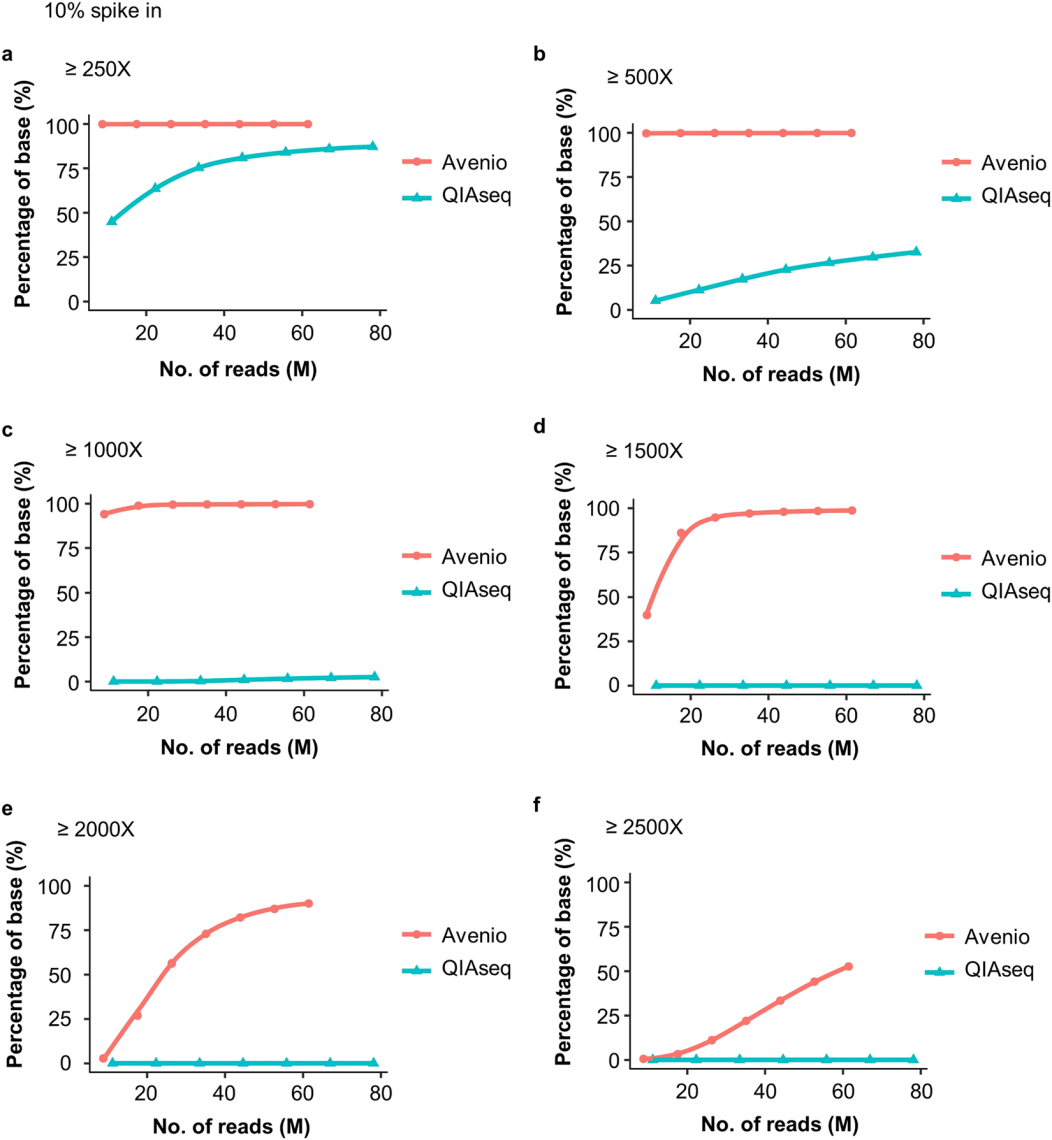
The percent of targeted bases covered at (**a**) ≥250-fold, (**b**) ≥500-fold, (**c**) ≥1000-fold, (**d**) ≥1500-fold, (**e**) ≥2000-fold and (**f**) ≥2500-fold read depth.

To assess the coverage of the targeted bases for each platform, the percentage of targeted bases in each panel that has been sequenced for at least 250, 500, 1000, 1500, 2000 and 2500X was quantified after removal of PCR duplication in the 10% cfDNA reference spike-in sample. Over 99% of targeted bases were covered ≥250X in Avenio while around 85% of bases were covered ≥250X in QIAseq with 80 million pair reads per sample (Fig. 6a). At all read counts and depth cut-offs, Avenio gave a higher unique coverage of targeted bases which may be a benefit from the smaller size of the panel (Fig. 6a–f).

The panel size of QIAseq is about five times (5.16X) larger than that of Avenio. To evaluate the performance of Avenio as if it is of the same size as QIAseq. We examined the relationship of sequencing depth and unique coverage of the targeted bases (250X, 500X, 750X, 1000X) after normalizing the Avenio sequencing reads by panel size ratio (5.16). After normalization, Avenio presented a higher unique coverage at low read depth threshold, ≥250X (Supplementary Fig. 1a). However, the unique coverage of Avenio decreased significantly especially at a higher read depth threshold (Supplementary Fig. 1a–d).

## Discussion

Users of commercialized cancer panels aim to detect clinically-relevant mutations for cancer profiling, response monitoring and therapy guiding. The work presented here is significant in helping the research, clinical and scientific community to make an informed decision about the platform best suit their applications. By comparing 2 commercialized cancer panels that use different target enrichment technologies, this study demonstrated the performance of both commercial kits for identifying highly diluted ctDNA in plasma. Using TCGA datasets consisting of 33 cohorts, both platforms were shown to enrich recurrent somatic mutations in common cancers. We have observed that the Avenio ctDNA Expanded panel was able to adequately cover most hotspots. Due to the use of an iterative algorithm to maximize the number of missense mutations per patient while minimizing the panel size during panel development. Thanks to its smaller panel size, it allows sequencing to a higher depth at a reasonable and affordable sequencing cost for the identification of diluted ctDNA in the plasma. On the other hand, QIAseq demonstrated excellent ability in targeting more mutations across all cancer types and has a higher patient coverage. The QIAseq panel showed higher patient coverage in some rare cancers and was designed to cover nearly 90% of patients at the early stages. Moreover, we have also demonstrated high sensitivity and concordance in the detection of ctDNA using both platforms. Therefore, the Avenio platform may be a better choice for applications to detect mutations in more common cancer types via sequencing at ultra-high depth while QIAseq suits applications aiming to have a broader spectrum of cancer types.

For the relative ease of application, Avenio requires 3 days for library construction while the library preparation with QIAseq takes only 1 working day. Although the turnaround time for hybridization-based enrichment workflow is often longer than its amplicon-based enrichment counterparts, the actual hands-on time for both panels is comparable. Furthermore, Avenio provides an end-to-end solution from cfDNA extraction to bioinformatic analysis. Therefore, Avenio may be more favorable in small labs or small scale of applications which do not have a bioinformatics team. In contrast, though the bioinformatic pipeline of QIAseq is open-sourced, it would require personnel with bioinformatics background to turn the out of sequencer raw data into annotated variants.

Both library preparation workflow tagged individual DNA fragment with UMI, 4 and 12 bp in length in Avenio and QIAseq respectively. Sequencing errors arisen from PCR amplification mis-incorporation, cluster amplification and sequencing account for a background level of 0.1–1% base mis-identification depending on sequencing platforms^28^. This error rate is within the same order of magnitude as most clinical variants in the highly heterogeneous cfDNA mixture and therefore hinders the identification of true rare variants. By uniquely tagging individual fragments with a UMI before amplification, variants with AF below 0.1% could also be detected^29–36^. The benefit of accurate quantification provided by UMI is evident with the high concordance between expected and observed AF of variants of higher AF. Our data showed that at AF less than 1%, the concordance correlation coefficient of Avenio is substantially higher than that of QIAseq. Together with a higher sensitivity of Avenio in detecting variants with low AF, Avenio outperforms QIAseq in the detection of ctDNA in plasma.

For the application on ctDNA profiling for early-stage asymptomatic screening, the tissue-of-origin would be needed to guide follow-ups. The Avenio bioinformatic pipeline annotates variants detected with TCGA data to provide information on the prevalence of certain mutations being detected in a specific cancer type. This may provide insight into the most probable tissue-of-origin based on the somatic mutation signature by a simple voting scheme.

The possibility of inferring tissue-of-origin by secondary analysis of genomic fragment ends, preserved by hybridization-enrichment library preparation but not its amplicon-based counterparts, is also an important factor for determining which enrichment technology and platform to be employed. The size distribution of cfDNA, which has a median of 166 bp corresponding to that of chromatosomes (nucleosome + linker histone, ~167 bp)^24^, has drawn speculations on the association of epigenetic landscape and DNA fragmentation pattern. Nucleosome occupancy protects DNA fragments from apoptotic nuclease digestion^37–39^. Recent studies have demonstrated that the DNA fragment ends retain information on the nucleosome occupancy and chromatin structure which highly correlates with the tissue-of-origin^40,41^. Moreover, the DNA fragment end pattern could be used not only to predict the tissue-of-origin for ctDNA profiling but also help eliminate false-positive caused by hemopoietic clonal mutations which is a major source of false-positive in ctDNA profiling. Library preparation workflow which ligates adapter to both ends of the fragment and enriches via hybridization preserves the genomic ends of the DNA fragments and thus the fragmentation pattern. In the case of amplicon-based target enrichment workflow, the use of gene-specific primers for enrichment causes the loss of information on the genomic coordinate of the fragment endpoints. Though the classification of tissue-of-origin based on fragmentation pattern inferred from fragment endpoints is still a field of on-going research, deep sequencing data generated by Avenio, which target enriches region of interest via hybridization with DNA baits, can be used not only for mutation identification but might also be secondarily processed to infer the possible tissue-of-origin^41^.

In summary, after the first identification of ctDNA in the circulation, the pursuit of developing a non-invasive, effective and affordable pan-cancer screening test is relentless. The use of ultra-deep sequencing with carefully designed and optimized pan-cancer panels and library construction workflow would allow direct measurement of the somatic changes in cancers. This study made a systemic comparison of the performance of Avenio ctDNA Expanded panel and QIAseq Human Comprehensive Cancer panel in ctDNA profiling. Both platforms demonstrated high patient coverage, sensitivity and concordance in the detection of clinically relevant variants with minimal cfDNA input. With a smaller panel, Avenio offers an excellent performance in detecting mutations in the hotspots of most common cancers with higher sensitivity and concordance. On the other hand, QIAseq outperforms by enabling detection in some relatively less common cancer types, therefore offering a true pan-cancer screening. These findings identified a unique niche for each of the commercially available panels.

## Methods

### Subjects

The panels were tested with the same starting DNA material originated from a 30-year-old healthy Han Chinese female. The subject does not demonstrate any pathological, histological or molecular sign of cancer. The subject was neither pregnant nor had received a blood transfusion within a month of commencement of the blood sampling of the study or diagnosed with autoimmune disease. The use of the specimen for the study and all experiments were conducted in accordance with the relevant guidelines and regulations approved by the Medtimes Medical Group Ethics Review Board. Written informed consent for the use of peripheral blood was obtained.

### Blood sampling

Blood samples were collected using Cell-Free DNA Collection tubes (Roche, Mannheim, Germany) by venipuncture and were stored at 4 ^o^C for not more than 24 hr before preprocessing. Blood samples were drawn carefully to avoid any hemolysis. Whole blood samples were centrifuged at 3,000 rpm for 10 min at 4 ^o^C followed by second centrifugation at 14,000 rpm for 10 min at 4 ^o^C to obtain cell-free plasma. Plasma was collected and stored in aliquots in −30 ^o^C until further analysis.

### Plasma DNA extraction and quantifications

DNA was extracted from 4 mL of plasma using cfDNA extraction kit (TIANGEN Biotech Co. Ltd., Beijing, China) with Kingfisher Duo Prime (Thermo Fisher Scientific, San Jose, CA) according to manufacturer’s instructions except for the following modification: 10 μL of beads was used for each 1 mL plasma extraction. For 4 mL plasma input, DNA was eluted into 130 μL of elution buffer and stored at −30 ^o^C. cfDNA was quantified using QuBit dsDNA HS Assay kit with QuBit 3.0 fluorimeter (Thermo Fisher Scientific, San Jose, CA).

### Library preparation with Avenio ctDNA Expanded kit

The kit was purchased from Roche (Mannheim, Germany). Sequencing libraries were prepared according to the manufacturer’s instructions with the following modifications: plasma cfDNA was extracted using cfDNA extraction kit (TIANGEN Biotech Co. Ltd., Beijing, China) instead of the Avenio ctDNA isolation kit included. Briefly, 15 ng of plasma cfDNA fragments were end-repaired, A-tailed, ligated with UMI-barcoded adaptors and amplified with PCR (12 cycles). The adaptor-ligated libraries were hybridized for 18 hr with biotinylated oligo DNA baits and enriched with streptavidin-conjugated magnetic beads. The target enriched libraries were further amplified for 15 cycles with PCR and were size-selected for an average fragment size of 350 bp. The library profile was analyzed with the Agilent 2100 Bioanalyzer (Agilent Technologies, Palo Alto, CA) and quantified using QuBit dsDNA HS Assay kit with QuBit 3.0 fluorimeter (Thermo Fisher Scientific, San Jose, CA) and qPCR with QIAseq Library Quant Assay Kit (QIAgen, Hilden, Germany).

### Library preparation with QIAseq Human Comprehensive Cancer panel

The kit was purchased from QIAgen (Hilden, Germany). Sequencing libraries were prepared according to the manufacturer’s instructions. Briefly, 15 ng of plasma cfDNA fragments were end-repaired, A-tailed, ligated with UMI-barcoded adaptors. The adaptor-ligated libraries were target enriched with PCR using a panel of loci specific primers (6 cycles). The target enriched libraries were further amplified for 21 cycles with PCR and were size selected for an average fragment size of 350 bp. The library profile was analyzed with the Agilent 2100 Bioanalyzer (Agilent Technologies, Palo Alto, CA) and quantified using QuBit dsDNA HS Assay kit with QuBit 3.0 fluorimeter (Thermo Fisher Scientific, San Jose, CA) and qPCR with QIAseq Library Quant Assay Kit (QIAgen, Hilden, Germany).

### Spike in dilution series

Dilution series were performed to assess the sensitivity and concordance of both panels in detecting and quantitating ctDNA. cfDNA references consist of frequent occurring mutations of AF quantified by ddPCR were spiked into circulating DNA of a healthy individual. A total of 15 ng of DNA containing 0.1 1, 10 and 50% of the cfDNA reference, comprised of expected AF from 0.00075–15%, was then used for library construction.

### Next-generation sequencing

Sequencing was performed on Illumina NextSeq 550. For a single sequencing run, a 4-multiplexed library was created by pooling the libraries, quantified using QuBit dsDNA HS Assay kit with QuBit 3.0 fluorimeter (Thermo Fisher Scientific, San Jose, CA) and qPCR with QIAseq Library Quant Assay Kit (QIAgen, Hilden, Germany), at an equal molar ratio. The multiplexed library was denatured and sequenced with NextSeq High Output kit 2 ×150 cycles paired.

### Read-count normalization

Total read count of all sequencing was normalized to 50–350 M reads with an increment of 50 M reads, by randomly drawing reads from the raw data. At each read depth, a triplicate of random drawings of reads was performed with a recorded random seed.

### Panel evaluation with TCGA variant data

Variant data of 10, 418 patients of 33 cancer types profiled by TCGA were downloaded from GDC Data Portal. Variant data called by 4 variant callers, Mutect2, Varscan2, MuSE and SomaticSniper were used to assess the average number of variants per patient covered by the cancer panels.

### Bioinformatic analysis

Two analysis pipelines for the cancer panel were compared. Raw sequencing data were analyzed with Avenio ctDNA analysis software (version 1.1.0) with default parameter settings for Expanded panel and QIAseq open sources analysis pipeline (version 2, dated May 29, 2018)^42^ with default parameter settings respectively.

### Data availability

Sequence data generated and analyzed during the current study are available in the Sequence Read Archive under accession number PRJNA577992 (https://www.ncbi.nlm.nih.gov/sra/PRJNA577992).

## Supplementary information

Supplementary information.
