# Supplementary material for: Comparison of Target Enrichment Platforms for Circulating Tumor DNA Detection
Source: Sci Rep. 2020 Mar 5;10:4124. doi: 10.1038/s41598-020-60375-x (PMC7057974; doi:10.1038/s41598-020-60375-x)
Supplement: Supplementary file 1 — Supplementary information. [file 41598_2020_60375_MOESM1_ESM.pdf]

Comparison of Target Enrichment Platforms for Circulating Tumor DNA Detection

So Ngo Lam[a, 1], Ying Chun Zhou[b, 1], Yee Man Chan[a], Ching Man Foo[a], Po Yi Lee[a], Wing Yeung Mok[a], Wing Sum Wong[a], Yan Yee Fung[a], Kit Yee Wong[a], Jun Yuan Huang[b], Chun Kin Chow[a,*]

[a] Department of Research and Development, Medtimes Molecular Laboratory Ltd., Hong Kong

[b] First Affiliated Hospital of Guangzhou University of Chinese Medicine, Guangzhou, Guangdong, China

[1] Both authors contributed equally to this work

* Correspondence and requests for materials should be addressed to Chun Kin Chow (isaac@medtimes.com.hk)

Supplementary data

Supplementary figure 1

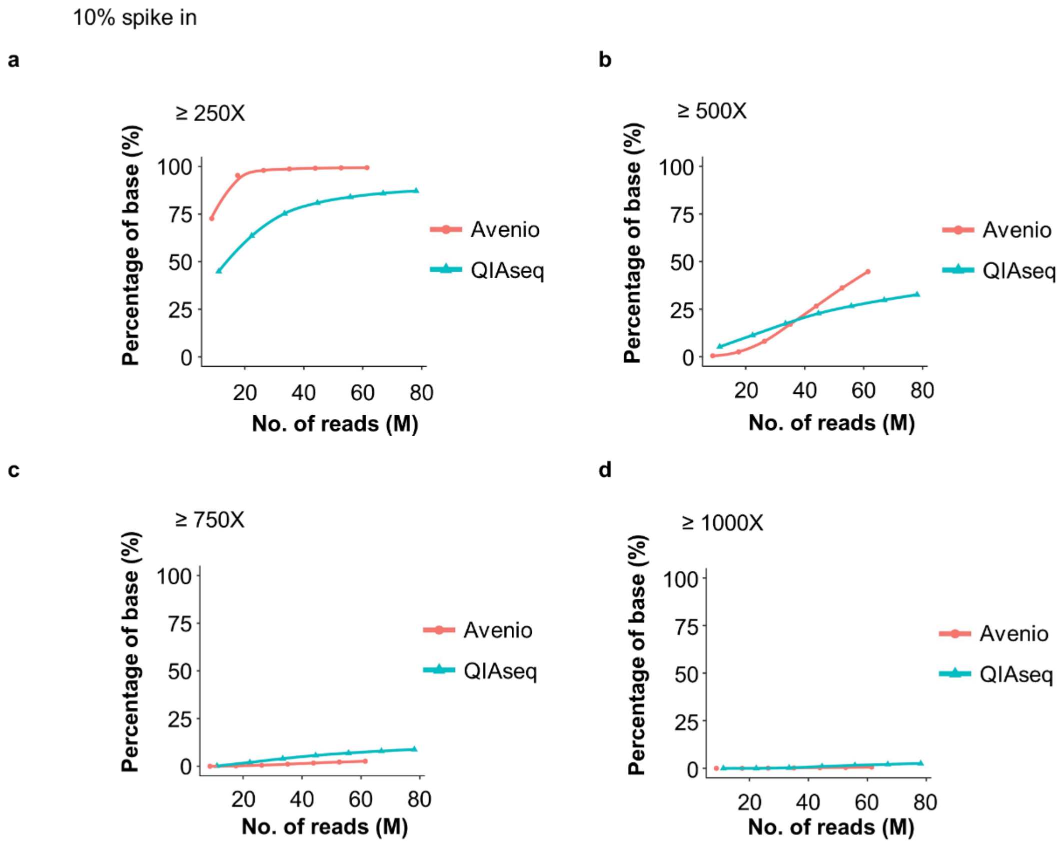

Supplementary Figure 1. Normalized percent of targeted bases covered at (a) ≥ 250-fold, (b) ≥ 500-fold, (c) ≥ 750-fold read depth and (d) ≥ 1000-fold read depth.
